# Simultaneity and Temporal Order Judgments Exhibit Distinct Reaction Times and Training Effects

**DOI:** 10.1371/journal.pone.0145926

**Published:** 2016-01-12

**Authors:** Nestor Matthews, Leslie Welch, Rebecca Achtman, Rachel Fenton, Brynn FitzGerald

**Affiliations:** 1 Department of Psychology, Denison University, Granville, Ohio, United States of America; 2 Cognitive, Linguistic, & Psychological Sciences, Brown University, Providence, Rhode Island, United States of America; University G. d'Annunzio, ITALY

## Abstract

A considerable body of sensory research has addressed the rules governing simultaneity judgments (SJs) and temporal order judgments (TOJs). In principle, neural events that register stimulus-arrival-time differences at an early sensory stage could set the limit on SJs and TOJs alike. Alternatively, distinct limits on SJs and TOJs could arise from task-specific neural events occurring after the stimulus-driven stage. To distinguish between these possibilities, we developed a novel reaction-time (RT) measure and tested it in a perceptual-learning procedure. The stimuli comprised dual-stream Rapid Serial Visual Presentation (RSVP) displays. Participants judged either the simultaneity or temporal order of red-letter and black-number targets presented in opposite lateral hemifield streams of black-letter distractors. Despite identical visual stimulation across-tasks, the SJ and TOJ tasks generated distinct RT patterns. SJs exhibited significantly faster RTs to synchronized targets than to subtly asynchronized targets; TOJs exhibited the opposite RT pattern. These task-specific RT patterns cannot be attributed to the early, stimulus-driven stage and instead match what one would predict if the limits on SJs and TOJs arose from task-specific decision spaces. That is, synchronized targets generate strong evidence for simultaneity, which hastens SJ RTs. By contrast, synchronized targets provide no information about temporal order, which slows TOJ RTs. Subtly asynchronizing the targets reverses this information pattern, and the corresponding RT patterns. In addition to investigating RT patterns, we also investigated training-transfer between the tasks. Training to improve SJ precision failed to improve TOJ precision, and vice versa, despite identical visual stimulation across tasks. This, too, argues against early, stimulus-driven limits on SJs and TOJs. Taken together, the present study offers novel evidence that distinct rules set the limits on SJs and TOJs.

## Introduction

What rules govern how we judge the relative timing of stimuli? This long-standing question has generated numerous experiments that required participants to judge either the simultaneity or the temporal order of stimuli. In principle, both simultaneity judgments (SJs) and temporal order judgments (TOJs) could depend simply upon the arrival time difference between two stimuli [1–3]. Despite the parsimony of this shared computation, however, SJs and TOJs appear to follow distinct rules. Evidence for distinct SJ- versus TOJ-rules comes from task-specific outcomes observed on two common relative-timing indices: the point of subjective simultaneity [4–8], and temporal precision [7, 9–13]. Notably, these distinct outcomes have been observed in experiments that vary the task between SJs and TOJs without varying the stimulation [7, 8]. This has inspired explanatory models that go beyond stimulus-driven factors and instead emphasize how SJs and TOJs differ in decision space. Accordingly, in the present study we consider a model of SJ and TOJ decision space, and test two of its predictions.

**Fig 1:**
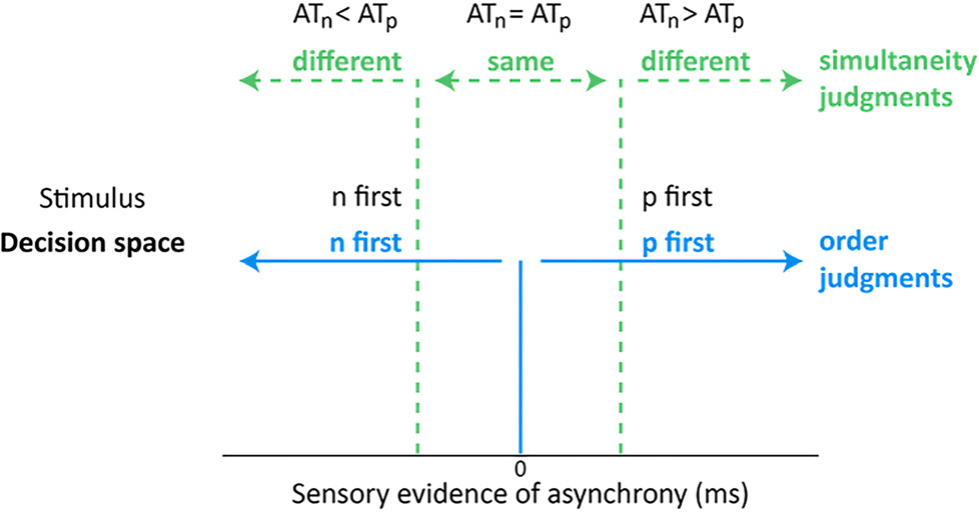
Decision space for relative timing judgments (Adapted from [14, 15]). The decision boundaries for “same”/“different” simultaneity judgments (SJs; dotted green vertical lines) differ from the decision boundary for “n first”/“p first” temporal order judgments (TOJs: solid blue vertical line at zero). On SJs, participants respond “same” when sensory evidence for the arrival time (AT) difference between stimulus “n” and stimulus “p” falls within the synchrony region (dotted green vertical lines; AT_n_ = AT_p_). Sufficiently asynchronous arrival times (AT_n_<AT_p_ or AT_n_>AT_p_) generate “different” SJ responses. On TOJs, participants respond “n first” when AT_n_<AT_p_, and “p first” when AT_n_>AT_p_.

Fig 1 schematizes a SJ and TOJ decision space, adapted from recent computational models of relative timing judgments [14, 15]. For illustration, we apply this decision space to a hypothetical experiment that requires either a SJ or a TOJ about two visual stimuli, "n" and p", on each trial. The figure's horizontal axis reflects on each trial the sensory evidence that the participant has regarding the arrival time (AT) difference between the stimuli: AT_n_—AT_p_. On SJ trials (color-coded green), the participant must report either that "n" and "p" occurred at the "same" time or that "n" and "p" occurred at "different" times. The model posits that AT differences falling within (AT_n_ = AT_p_) and outside (AT_n_<AT_p_, or AT_n_>AT_p_) the synchrony region (bounded by dotted green vertical lines) respectively generate "same" and "different" SJ responses. On TOJ trials (color-coded blue), the participant must report either that "n" appeared first or that "p" appeared first. The model posits that negative (AT_n_ < AT_p_) and positive (AT_n_ > AT_p_) AT differences respectively generate "n first" and "p first" TOJ responses.

The decision space schematized in Fig 1 suggests a novel prediction: distinct reaction-time patterns for SJs and TOJs. To understand why, recall that reaction times (RTs) increase with decisional uncertainty [16–19]. Decisional uncertainty increases as the sensory evidence of asynchrony approaches a decision boundary for a given relative-timing task. On the SJ task, uncertainty would be greatest–and RTs presumably largest (slowest)–when the sensory evidence of asynchrony reaches either boundary for the synchrony region (demarcated in Fig 1 by dotted green vertical lines). This contrasts sharply with the smaller (briefer) RT one would expect on SJ trials generating evidence of asynchrony near zero ms (Fig 1, solid blue vertical line), i.e., the strongest case for a “same” response. Operationally, one can track these hypothesized uncertainty fluctuations via an RT ratio, defined as

RTtosynchronizedstimuli/RTto±thresholdasynchrony

**Fig 2:**
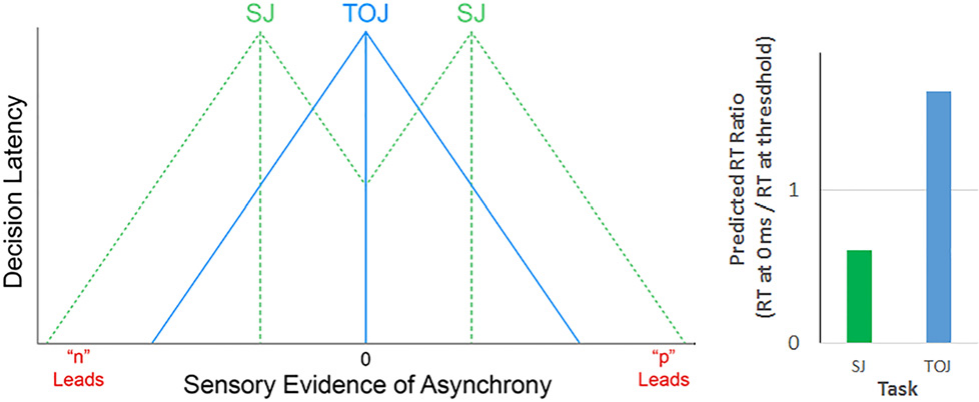
Task-specific decision latencies generate task-specific RT ratio predictions. The left panel adds decision latencies (y-axis) to the SJ (dotted green) and TOJ (solid blue) vertical decision boundaries, copied here from Fig 1. One would expect these task-specific decision boundaries to generate task-specific decision latencies (cartooned here via triangular trend lines) because decision latency increases with decisional uncertainty [16–19], which increases with proximity to a decision boundary. Specifically, one would expect larger SJ decision latencies (bimodal, dotted green triangular trend lines) near the synchrony region’s boundaries (dotted green vertical lines) than when the sensory evidence for asynchrony nears zero (solid blue vertical line). TOJ decision latencies (unimodal, solid blue triangular trend lines) would be expected to exhibit the opposite pattern. The triangular functions cartooned in the left panel emphasize the peak points at which SJs (dotted green) and TOJs (solid blue) differ in decision latency. The actual functions would be modeled more plausibly by Gaussian, Poisson, or other smoothly changing functions -empirically determinable by finely sampling the asynchrony space. The left panel’s task-specific decision latencies generate the right panel’s correspondingly task-specific RT ratio predictions. The RT ratio entails dividing the RT at 0 ms (left panel’s solid blue vertical line) by the RT at threshold (left panel’s dotted green vertical lines, presumptively the synchrony region’s boundaries). One would expect these RT ratios to be less than one for SJs (right panel, green column) and greater than one for TOJs (right panel, blue column).

An RT ratio *less than 1* would be predicted for SJs. This follows because one would expect low uncertainty for synchronized stimuli (generating small RTs for the ratio's numerator), and high uncertainty for threshold asynchronies (generating large RTs for the ratio's denominator). Conversely, under the same conditions an RT ratio *greater than 1* would be predicted for TOJs, which have an uncertainty pattern complementary to that for SJs. Specifically, Fig 1 implies high TOJ uncertainty for synchronized stimuli (generating large RTs for the ratio's numerator), and lower TOJ uncertainty for threshold asynchronies (generating smaller RTs for the ratio's denominator). Overall, to the extent that the performance limits on SJs and TOJs originate from a decision space akin to that schematized in Fig 1, one would expect the tasks to exhibit distinct reaction-time ratios. Fig 2 schematizes how distinct decision latencies (left panel) for SJs (dotted green triangular trend lines) and TOJs (solid blue triangular trend lines) generate these task-specific reaction-time ratio predictions (right panel).

**Fig 3:**
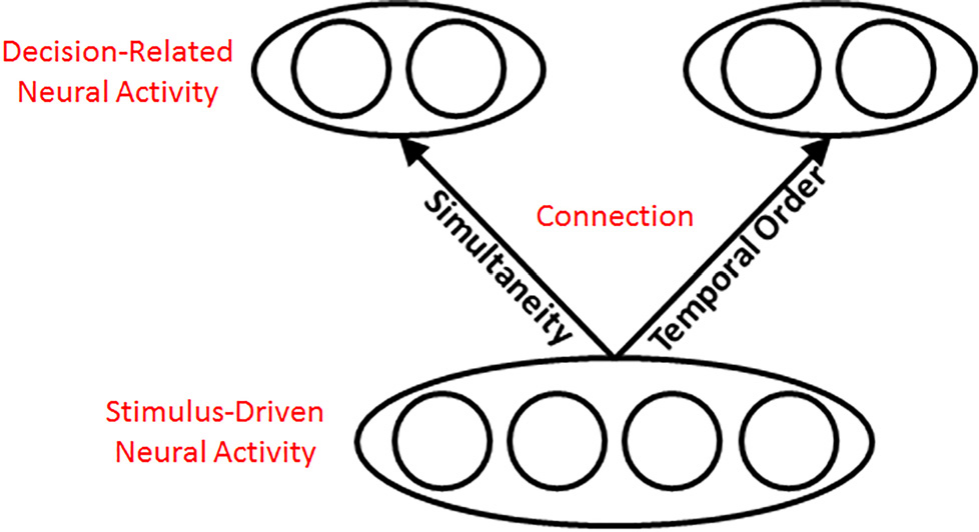
Localizing Training Effects (Adapted from [20]). The schematic allows for testing the diverging predictions from positing shared versus distinct sources of neural activity that limit SJ- and TOJ-precision. If decision-related neural activity and/or the “connection” limited SJ- and TOJ-precision, training on one task would not improve precision on the other, given identical visual stimulation. By contrast, if shared stimulus-driven neural activity at an early visual stage limited SJ- and TOJ-precision, training on one task would improve precision on the other, given identical visual stimulation.

A second prediction similarly originates from the possibility that decisional factors set a limit on SJs and TOJs. Here though, the limit pertains not to reaction time but rather to SJ and TOJ *precision*–the briefest stimulus asynchronies that participants can judge reliably. The limits on SJ and TOJ precision can be contextualized by considering the limitations associated with various visual pathway stages. In any biological system, no stage can have infinite precision. As a result, the precision of an SJ or TOJ can never exceed the least precise stage in the sequence. Stated differently, the least precise stage in the sequence of visual pathway stages sets the limit on SJ and TOJ precision. Intriguingly, one can draw informed conclusions about precision-limiting visual pathway stages by behavioral experiments that analyze whether learning transfers from trained to untrained tasks. Fig 3 schematizes a general framework for such an analysis. Here, we will use Fig 3 to develop the distinct, testable behavioral predictions that permit inferences about precision-limiting visual pathway stages. We postpone to the Discussion speculation about specific brain areas and neural synchrony patterns plausibly associated with the stimulus-driven, connection, and decision-related stages schematized in Fig 3.

If the limit on SJ and TOJ precision arose from task-specific decisions–schematized in Fig 3‘s top ovals–one would expect no training-transfer between tasks. That is, training to improve temporal precision on one task would not benefit temporal precision on the other task. One would similarly expect no training-transfer between tasks if the limit on SJ and TOJ precision arose from task-specific neural connections between decision-related and stimulus-driven neural activity–schematized in Fig 3‘s task-specific “connection” arrows. A very different outcome would be expected, however, if the limit on SJ and TOJ precision arose from stimulus driven-neural activity shared by the two tasks–schematized in Fig 3‘s bottom oval. In this case, training on either task would improve precision on the other. In short, Fig 3 provides a framework to localize the visual information stage that sets the limit on SJ and TOJ precision.

**Fig 4:**
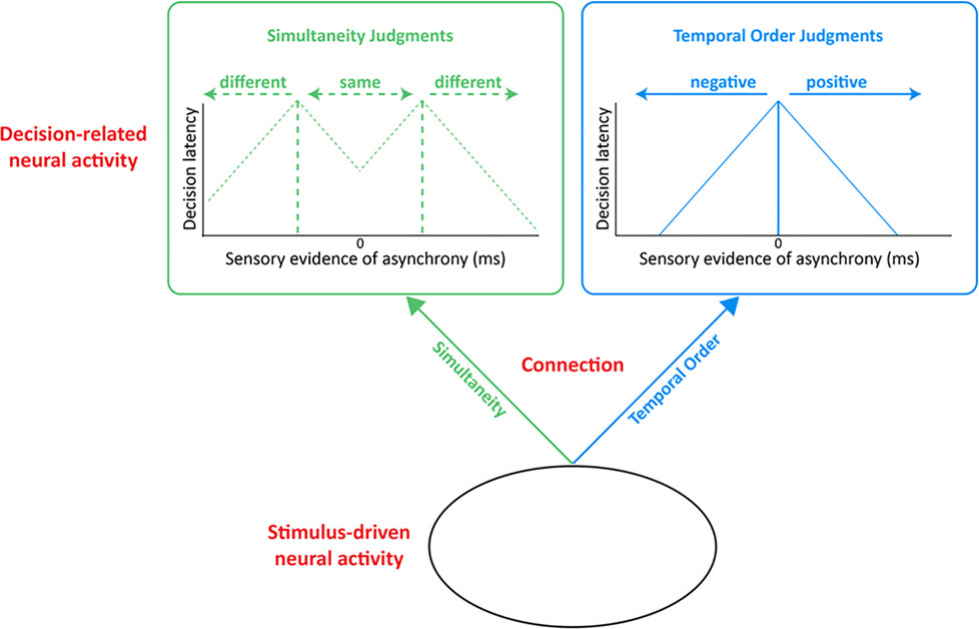
Conceptual framework for the present study. This model synthesizes Fig 1‘s decision space (adapted from [14, 15]), Fig 2‘s decision latencies, and Fig 3‘s schematic for localizing training effects (adapted from [20]). The model’s RT ratio predictions arise from the temporal boundaries within the SJ (dotted green) and TOJ (sold blue) decision spaces (top rectangles). The model’s training-transfer predictions about temporal precision arise from the task-specific (decision-related and “connection” (arrows) stages) versus shared (stimulus-driven stage (bottom oval)) nature of each stage. Table 1 summarizes the model’s predictions.

Figs 1, 2 and 3 come together in Fig 4, which depicts the present study's conceptual framework. This synthesized framework comprises distinct SJ (green) and TOJ (blue) decision spaces (boxes), and distinct SJ and TOJ “connections” (arrows) from stimulus-driven neural activity shared by the two tasks (bottom oval). By detailing decision-boundary locations, decision latencies, *and* the decision stage's relationship to the earlier "connection" and stimulus-driven stages, the model makes clear predictions about factors that govern SJs and TOJs. Specifically, if decision-boundaries set the limit on SJs and TOJs, one would predict the task-specific RT ratios described above, and that training on either task would not improve precision on the other. One would similarly predict no training-transfer if the limit on SJs and TOJs arose from the task-specific “connection” stage. However, because the task-specific “connection” contains no information about decision boundaries, this stage–unlike the decision stage–provides no basis for predicting task-specific RT ratios. Even greater predictive divergence would be expected if the limit originated in the stimulus-driven stage–a possibility that generates predictions directly opposite to those of a decision-stage limit. That is, the stimulus-driven stage (like the “connection” stage) contains no information about decision boundaries, and consequently provides no basis for predicting task-specific RT ratios. Additionally, because both tasks “inherit” the same stimulus-driven neural activity, training that improves temporal precision in this early phase would transfer to SJ- and TOJ-precision alike, if the stimulus-driven stage set the limit. Table 1 summarizes these model predictions.

**Table 1:** Summary of model predictions tested in the present study. See text for the basis of each prediction.

Limiting Visual Stage	Predicts Task-Specific RT Ratios?	Predicts Transfer-of-Training For SJ and TOJ Precision?
Decision-Related	Yes	No
Connection	No	No
Stimulus-Driven	No	Yes

We tested these model predictions in the present study by varying the tasks between SJs and TOJs while maintaining identical visual stimulation. The visual stimulation comprised dual-stream Rapid Serial Visual Presentation (RSVP) displays containing two targets–a red letter, and a black number–embedded within streams of black-letter distractors. This choice of stimuli offered two advantages. First, it allowed us to assess SJs and TOJs within a single modality (vision) while avoiding motion artifacts that often arise from asynchronized unimodal stimuli. Second, the exact same RSVP stimuli recently revealed distinct Points-of-Subjective-Simultaneity (PSSs) for SJs and TOJs [8]. If these stimuli were to also generate distinct RT ratios *and* no training-transfer on our SJ- and TOJ-precision measures, there would be compelling evidence that decision-related factors set the limit on these two relative-timing tasks.

## Materials and Methods

### Participants

Denison University's Human Subject Committee approved all experiments in this study, which we conducted with the understanding and written consent of each participant. Participants comprised 72 Denison University undergraduates who each reported normal or corrected vision. All were naïve regarding the research purposes.

### Apparatus & Stimuli

We used the apparatus and stimuli described in Matthews et al. (2013), but for completeness we repeat here the apparatus and stimulus details. The stimuli in that study strongly resembled those that Verleger et al. [21, 22] employed for measuring event-related potentials during a target identification task.

Experiments were conducted on Dell OptiPlex780 desktop computers, each with a Microsoft Windows 7 Enterprise operating system. SuperLab 4.5 presentation software (Cedrus) controlled 17-in (43.18-cm) flat screen Dell 2009W displays, each with a 60 Hz vertical refresh rate and 1680 × 1050 spatial resolution. Although head position was not stabilized, viewing distance was typically 57 cm from the monitor.

**Fig 5:**
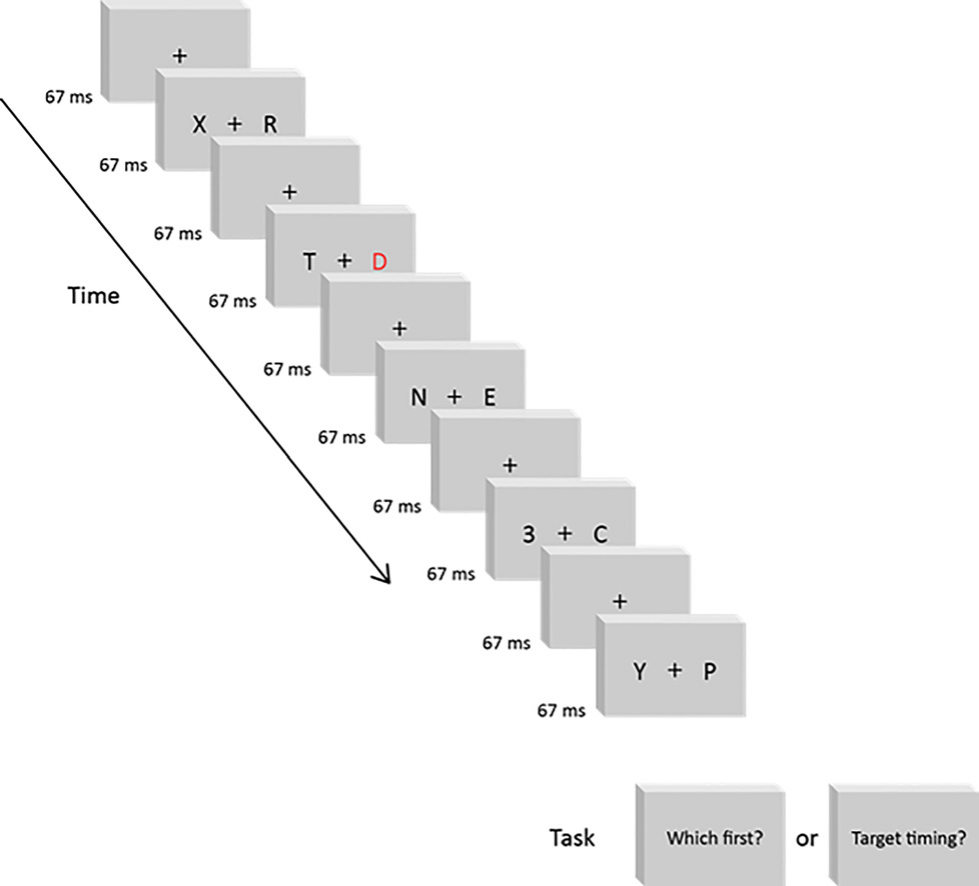
RSVP Sequence. Each RSVP sequence contained 20 bilaterally presented stimulus pairs that included black-letter distractors and two targets–a red letter, and a black number. The two targets were presented in opposite lateral hemifields, either synchronously or at various asynchronies. The sequence above schematizes a RVF red letter (“D”), preceding a LVF black number (“3”) by 268 msec. Participants judged the targets’ temporal order (“Which first?”: ‘letter first’ versus ‘number first’) in one session and the targets’ simultaneity (“Target timing?”: ‘same’ time versus ‘different’ times) in a separate session. For the schematic above, the correct temporal order and simultaneity responses respectively correspond to “letter first” and “different”. S1 Movie shows a sample RSVP trial.

The stimulus on each trial was a dual-stream RSVP sequence (Fig 5 and S1 Movie). Each sequence comprised forty 15 Hz frames (67 ms / frame; 2.667 sec total), containing a black fixation cross (0.5 x 0.5 deg) centered in a white surround. Across each forty-frame sequence, we presented twenty bilateral stimulus pairs, one on each odd-numbered frame. Even-numbered frames contained only the fixation cross. Consequently, visual *transients* occurred at 15 Hz (every 67 ms) but new stimulus *information* occurred at 7.5 Hz (every 133 ms). Within each new stimulus pair, 3.5 deg separated (center-to-center, horizontally) the fixation cross from each laterally flanking stimulus. The flanking stimuli were either Arabic numbers or capitalized Latin letters (Calibri font, stroke-width 0.02 deg) extending 2.0 deg vertically and a maximal 2.0 deg horizontally.

Each RSVP sequence contained two targets (one in each lateral hemifield) and 38 distractors (19 in each lateral hemifield). One of the targets was a black number, randomly selected on each trial from the set 1, 2, 3, 4, 5, and 6. The other target was a red letter, randomly selected on each trial from the set D, F, G, J, K, and L. The black number and red letter targets occurred equally often in each lateral hemifield, randomly across trials. The distractors comprised all other letters, randomly sequenced, and always presented in black.

### Tasks

Simultaneity judgments required participants to report whether the red letter target and the black number target flashed at the “same” time, or at “different” times. Temporal order judgments required participants to report which of the two targets flashed first, “letter” versus “number”. We encouraged participants to maximize accuracy, and did not mention reaction time. Participants responded on a standard computer keyboard, with no restrictions on which finger or hand to use.

### Procedure

Visual stimulation remained identical across tasks. To minimize task confusion, each participant performed only one of the two tasks per daily session: either SJs only, or TOJs only. Within each daily session, each participant completed 22 practice trials, followed by five 120-trial blocks (600 trials for analysis). Each 120-trial block comprised 40 synchronized trials and 80 asynchronized trials. The 80 asynchronized trials comprised four instances each of twenty experimental conditions. These comprised five target asynchronies (133, 267, 400, 533, and 667 ms) crossed with two letter-target-hemifield configurations (LVF-letter versus RVF-letter) crossed with two target orders (letter leading versus letter lagging). Among the 40 synchronized trials, half contained LVF-letter (RVF-number) targets and half contained RVF-letter (LVF-number) targets. All trials contained at least one target in the 11^th^ stimulus pair, which occurred 1.333 sec into the RSVP sequence. All conditions were randomized anew within each 120-trial block.

On the simultaneity task, participants were informed initially–and subsequently reminded between trials blocks–that asynchronized and synchronized trials would occur in a 2:1 ratio. On the temporal order task, participants were informed that “letter first” and “number first” trials would occur equally often. To maintain motivation on each task, immediate visual feedback identified each response as correct or incorrect. “Letter first” and “number first” responses on TOJ trials with synchronized targets were not objectively classifiable as correct or incorrect. Consequently, half of the synchronized TOJ trials were pre-designated arbitrarily as “letter first” and half as “number first” to avoid biasing participants’ responses with feedback.

### Experimental designs

#### Decision-boundaries experiment

We tested the predictions that arise from the task-specific decision boundaries schematized in Fig 4. This entailed comparing reaction times for asynchronies close to the boundaries with reaction times for asynchronies farther from the boundaries. The experiment comprised a single within-participants independent variable, Task (SJ vs TOJ). SJ and TOJ tasks occurred on different days, counter-balanced across participants. Forty-seven participants in the decision-boundaries experiment satisfied the inclusion criteria (described below).

#### Training-transfer experiment

**Fig 6:**
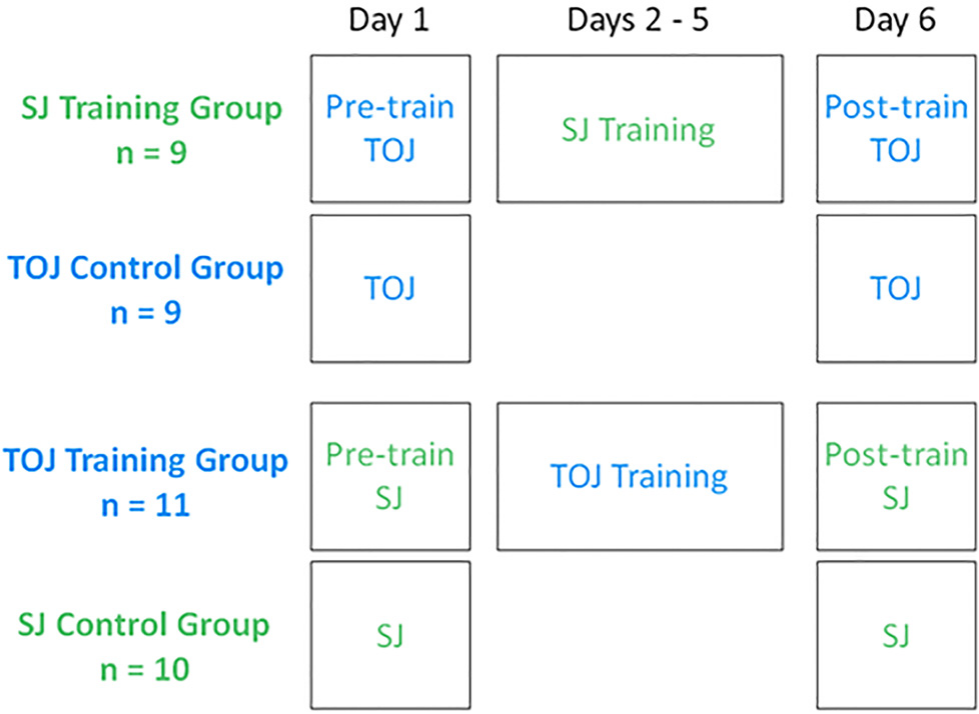
Daily schedule for the training-transfer experiment. SJ and TOJ sessions appear in color-coded green and blue lettering, respectively. Training group participants trained on days 2–5. Control group participants did not perform either task between pre- and post-training sessions.

We evaluated training-transfer using a mixed-factorial design, comprising the independent variables “Group” and “Day” schematized in Fig 6. Participants in the SJ training group and the TOJ training group returned to complete a total of six daily sessions. We used data from days 1 and 2 for these participants to contribute to the above-described decision-boundaries analysis. Note that the SJ Training Group and TOJ Training Group performed complementary task sequences (SJs versus TOJs) across sessions. Participants in the control groups completed *either* TOJs only (TOJ control group) *or* SJs only (SJ control group), across two daily sessions. This allowed us to quantify simple acclimation to each task, absent any influences that might arise from performing the other task. The sample size (n) for each group in Fig 6 reflects the number of participants who satisfied the inclusion criteria (described below).

### Data Analysis

#### Reaction time

To determine whether the pattern of reaction times depended on temporal task, we measured the RT ratio described in the Introduction:

RTat0ms/RTat±133ms

Specifically, for each participant and session the components of the RT ratio comprised the median RTs at the 0 ms (200 trials) and ±133 ms (80 trials) asynchronies. We chose the ±133 ms asynchronies because they mostly closely matched the asynchrony thresholds that we empirically observed in a prior study with the same stimuli [8]. As noted in the Introduction, one would expect lower SJ than TOJ RT ratios if reaction times depended critically on the task-specific decision boundaries in Fig 4. We assume that the RTs within each RT ratio reflect the sum of the durations associated with sensory, motor, and decisional factors [23, 24]. Because sensory information remained identical across tasks, and each task required only a simple binary motor response on a standard keyboard, significant task differences in RT ratios plausibly reflect task-specific decision latencies (Fig 2).

#### Temporal precision

For each SJ session, we assessed each participant’s temporal precision using standard signal detection procedures to determine *d′* for each target asynchrony [25]. Computationally, *d′* corresponds to (zHits)–(zFalse Alarms) with *d′* = 0.67 corresponding to the nonbiased 75% discrimination threshold. Operationally, hits and false alarms occurred when participants made “different” responses to asynchronized and synchronized targets, respectively.

For each TOJ session, we assessed each participant’s temporal precision by constructing a psychometric function. Each psychometric function’s ordinate reflected the proportion of “letter first” responses. The abscissa comprised target asynchronies ranging between -667 ms (letter lagging) and +667 ms (letter leading), in 133 ms steps. We used a least-squares procedure to fit the data with a sigmoid of the form

1/(1+exp(−K(X−Xo))

where K and Xo determined the slope and midpoint of the sigmoid, respectively. The best-fitting sigmoid correlated well with each participant's responses (mean correlation coefficient (r) = 0.981, median = 0.992, range = 0.849–0.999). From the best-fitting sigmoid we interpolated the 75% just noticeable difference–a nonbiased TOJ discrimination threshold reflecting half the stimulus change (target asynchrony) required to alter the response rate from 0.25 to 0.75. This corresponds with *d’* = 0.67.

#### Inclusion / exclusion criteria

We excluded data from ten (14% of 72) participants who, on one or more daily sessions, failed to perform above chance levels and/or exhibited thresholds requiring extrapolation beyond the tested asynchrony range (±667 ms). Two of these ten participants failed on SJs, eight failed on TOJs. The remaining 62 (86% of 72) participants performed well enough to avoid those exclusion criteria. Their data appear in the Results. Some of these participants contributed data to both our reaction-time analysis and our temporal-precision analysis. Other participants contributed data to only one of these two analyses. As a result, the number of participants varies across the statistical analyses reported here. For clarity, S1 List contains a participant-list for each of the analyses. For completeness, S1–S5 Data contain the raw data from all 72 participants.

## Results

### Decision-boundaries experiment

**Fig 7:**
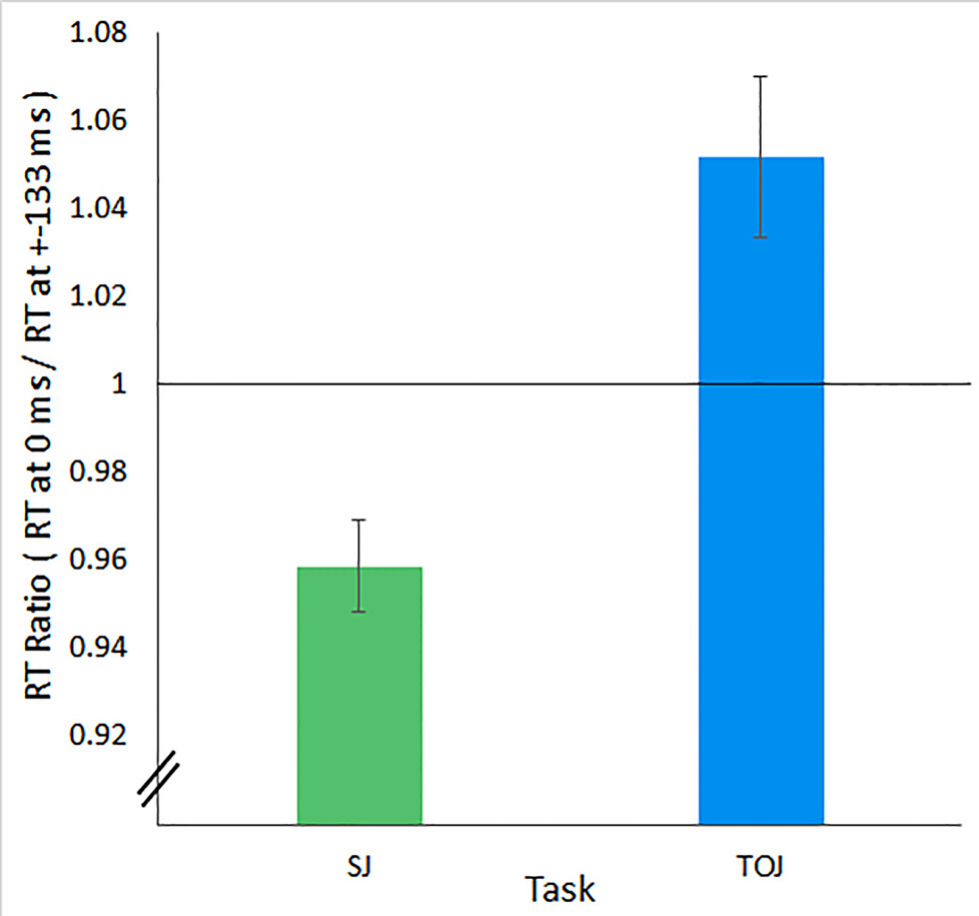
Reaction Time Ratios. Reaction time ratios (RT at 0 ms / RT at ±133 ms) for simultaneity judgments (SJs; green bar) and temporal order judgments (TOJs; blue bar) fell significantly below and significantly exceeded 1.0, respectively. Error bars reflect ±1 SEM (n = 47). Note that the vertical axis does not start at zero.

Fig 7 shows our primary finding–a significantly lower reaction time (RT) ratio for SJs than for TOJs (t(46) = 4.347, p<0.001, _p_η^2^ = 0.291). Indeed, the RT ratio fell significantly below 1.0 for SJs (one sample t-test, t(46) = 91.490, p<0.001, _p_η^2^ = 0.994), and significantly exceeded 1.0 for TOJs (one sample t-test, t(46) = 57.607, p<0.001, _p_η^2^ = 0.986). This pattern was largely, though not perfectly consistent across participants; forty (85%) of the 47 participants meeting the inclusion criteria for this analysis exhibited greater TOJ than SJ RT ratios (binomial test p<0.001). These within-participant task differences occurred even though visual stimulation remained identical across tasks, and despite counter-balancing the task order. The data match what one would expect if RT increased with uncertainty near the task-specific decision boundaries schematized in Fig 4.

**Fig 8:**
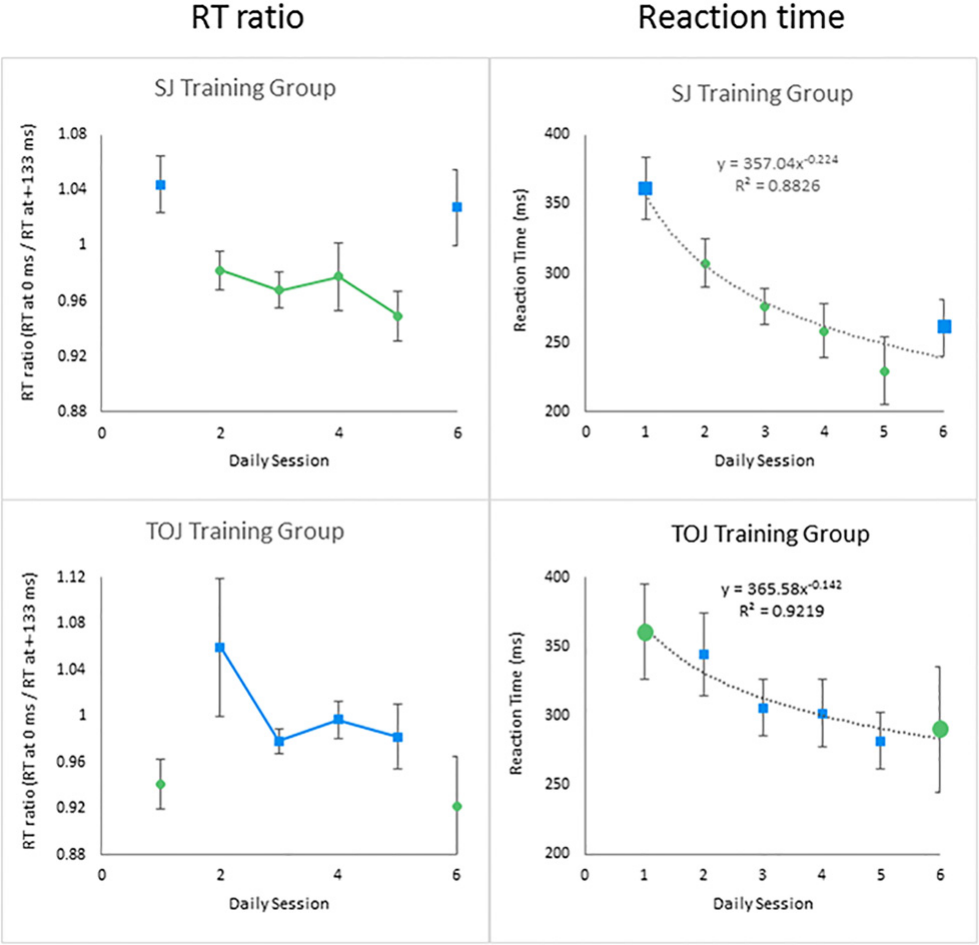
Reaction Time Ratios and Reaction Times from the training-transfer experiment. Each group performed either SJs only (green circles) or TOJs only (blue squares) within each of the six daily sessions. Across sessions, the two training groups performed complementary task sequences (SJs versus TOJs), which generated U-shaped (SJ Training Group, top left panel) and inverted U-shaped (TOJ Training Group, bottom left panel) trends in RT ratios. Within each group, RT ratios for TOJs consistently exceeded those for SJs. Unlike these quadratic trends in RT *ratios* (left panels), the right panels show that *reaction times* decreased significantly across sessions regardless of task or group (power function fits to the data p<0.01). Error bars reflect ±1 SEM (SJ Training Group n = 9; TOJ Training Group n = 11).

Further evidence for task-specific RT ratios came from our training-transfer experiment (Fig 8, left panels). Across the experiment's six daily sessions, the SJ training group exhibited an RT ratio pattern complementary to that of the TOJ training group. Specifically, the SJ training group's RT ratios formed a U-shape, with higher RT-ratios occurring on days 1 and 6 (TOJs) than on days 2–5 (SJs) (Fig 8, top left panel). Conversely, the TOJ training group's RT ratios formed an *inverted* U-shape, with lower RT-ratios occurring on days 1 and 6 (SJs) than on days 2–5 (TOJs) (Fig 8, bottom left panel). A mixed 6x2 (Day-by-Task) ANOVA confirmed these distinct patterns via a significant quadratic trend in the Day-by-Task interaction (F(1,18) = 8.248, p = 0.01, _p_η^2^ = 0.314). One can intuit that interaction by mentally differencing the SJ and TOJ RT ratios across the six daily sessions, and noting the U-shaped (or *inverted* U-shaped) pattern of difference scores. Stated differently, each group exhibited higher TOJ than SJ RT ratios across daily sessions, despite identical visual stimulation. The observed data pattern matched what one would predict if reaction times increased with uncertainty near the task-specific decision boundaries schematized in Fig 4.

The task-*specific* pattern of RT *ratios* (Fig 8's left panels) contrasts sharply with the task-*independent* pattern of *reaction times* (Fig 8's right panels). Indeed, for each training group, reaction times decreased significantly (p<0.01) across the six daily sessions despite task switching on days 1-to-2 and days 5-to-6. The significant decrease in reaction times evinces learning curves for the RSVP stimuli, which many participants spontaneously described as challenging upon initial exposure. Some participants also spontaneously described the days 5-to-6 task transition as challenging–a subjective claim supported by the modest RT increase observed across the corresponding days. Indeed, some participants failed to meet the inclusion criteria only because of poor performance on day 6.

### Temporal Precision

**Fig 9:**
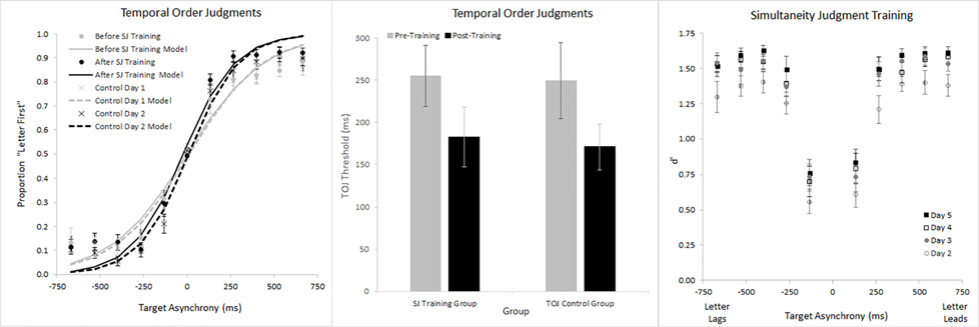
Temporal precision for the SJ Training Group and the TOJ Control Group. The left panel shows Temporal Order Judgment psychometric functions for both groups before (gray symbols) and after (black symbols) training. The central panel shows Temporal Order Judgment thresholds (*d’* = 0.67) for both groups before (gray bars) and after (black bars) training. Threshold improvement for both groups approached significance, but there was no Session x Group interaction. These data show that training on the SJ task did not improve precision on the TOJ task. The right panel shows Simultaneity Judgment performance (*d’*) across training days 2–5, and that performance did improve. Error bars reflect ±1 SEM (SJ Training Group n = 9, TOJ Control Group n = 9).

In addition to analyzing RTs, we also analyzed temporal precision. Fig 9 shows temporal precision for the SJ Training Group (circles) and the TOJ Control Group (X’s) in our training-transfer experiment. The figure’s left panel reveals modestly steeper psychometric functions for the second TOJ session (black symbols) than for the first (gray symbols). These psychometric functions generated the corresponding TOJ thresholds in Fig 9‘s central panel, where the first and second TOJ sessions again appear respectively in gray and black shading. TOJ precision improved (thresholds decreased) on the second session by margins that approached significance and explained 36.8% of the SJ Training Group’s variance (t(8) = 2.158, p = 0.063), and 34.5% of the TOJ Control Group’s variance (t(8) = 2.051, p = 0.074). Critically, the Session-by-Group interaction fell far short of significance (F(1,16) = 0.015, p = 0.905, _p_η^2^ = 0.001). This null interaction disconfirms training-transfer from SJs to TOJs. Indeed, the comparable TOJ improvement across the two groups could reflect simple acclimation; by the second TOJ session *all* participants may have become familiar with the RSVP stimuli, regardless of SJ training. Notably, the null effect of SJ training on TOJs occurred even though visual stimulation across tasks remained identical, and despite significant SJ (*d’*) improvements over the four SJ training sessions (Days 2–5; Fig 9‘s right panel; Session Linear Trend (F(1,8) = 35.934, p<0.001, _p_η^2^ = 0.818)). Collectively, Fig 9‘s data *argue against the possibility that shared stimulus-driven factors set* the temporal-precision-limit on SJs and TOJs.

**Fig 10:**
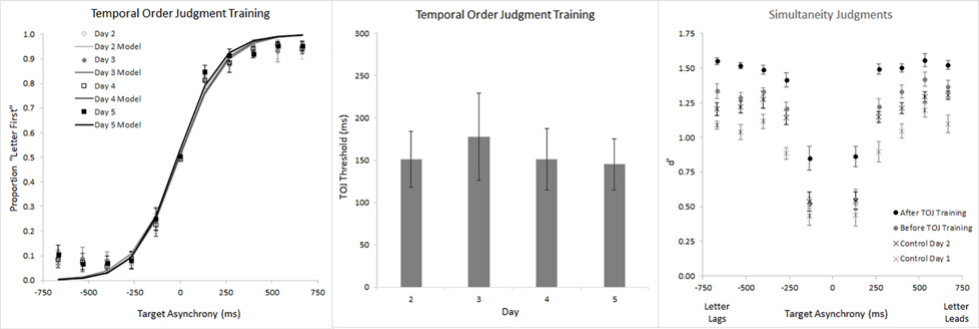
Temporal precision for the TOJ Training Group and the SJ Control Group. The left panel shows Temporal Order Judgment psychometric functions for the TOJ Training Group across four days of training. The central panel shows Temporal Order Judgment thresholds (*d’* = 0.67) derived from the psychometric functions in the left panel. Across the four training sessions, thresholds remained intermediate to the briefest two (of five) asynchronies tested. The right panel shows Simultaneity Judgment performance (*d’*) for the TOJ Training Group and SJ Control Group before (gray) and after (black) training. SJ precision (*d’*) improved on the second session by margins that reached significance and explained 34.4% of the TOJ Training Group’s variance, and 41.9% of the SJ Control Group’s variance. Critically, the Session-by-Group interaction fell far short of significance. These data show that training on the TOJ task did not improve precision on the SJ task. Error bars reflect ±1 SEM (TOJ Training Group n = 11, SJ Control Group n = 10).

Fig 10 provides further evidence for independence between the visual information stages that limit temporal-precision on SJs and TOJs. To appreciate this point first consider Fig 10‘s right panel (Simultaneity Judgments graph), which shows the TOJ Training Group (circles) and SJ Control Group (X’s) across the first (gray) and second (black) SJ sessions. SJ precision (*d’*) improved on the second session by margins that reached significance and explained 34.4% of the TOJ Training Group’s variance (t(10) = 2.29, p = 0.045), and 41.9% of the SJ Control Group’s variance (t(9) = 2.55, p = 0.031). Critically, the Session-by-Group interaction fell far short of significance (F(1,19) = 0.199, p = 0.66, _p_η^2^ = 0.010). This null effect disconfirms training-transfer from TOJs to SJs. Indeed, the comparable SJ improvement across the two groups could reflect simple acclimation; by the second SJ session *all* participants may have become familiar with the RSVP stimuli, regardless of TOJ training. Notably, the null effect of TOJ training on SJs occurred even though visual stimulation across tasks remained identical, and despite good performance over the four TOJ training sessions. Specifically, psychometric functions (Fig 10‘s left panel) and TOJ thresholds (Fig 10‘s center panel) on days 2–5 reveal just-noticeable-differences consistently between 150 and 200 ms–values intermediate to the briefest two (of five) asynchronies tested. Collectively, Fig 10‘s data disconfirm a model in which shared neural events set the temporal-precision-limit on SJs and TOJs.

## Discussion

The present study addresses factors governing relative timing judgments. Participants judged either the simultaneity or temporal order of red-letter and black-number targets presented in opposite lateral hemifield streams of black-letter distractors. For simultaneity judgments (SJs), we found significantly faster reaction times to synchronized targets than to subtly asynchronized targets. This pattern reversed when we presented the same stimuli to the same participants but switched the task to temporal order judgments (TOJs). These task-specific patterns match what one would predict if reaction time slowed as the sensory evidence for asynchrony approached the SJ and TOJ decision boundaries schematized in Fig 4 (boxes). Stated differently, our data imply that the decision stage played a larger role than did either the “connection” or stimulus-driven stages (Fig 4‘s arrows and bottom oval, respectively) in determining SJ and TOJ reaction times. We also found that SJ-training did not improve TOJ precision, and that TOJ-training did not improve SJ precision. Because we observed this absence of training-transfer while visual stimulation remained identical across tasks, the data argue against the possibility that the limit on SJ and TOJ precision originated at the stimulus-driven stage. Instead, the present training-transfer data indicate that the limit on SJ and TOJ precision originated in the “connection” or decision stages schematized in Fig 4. As we noted in the Introduction, however, only the decision stage contains the information necessary to also predict the observed task-specific RT patterns. Overall then, the present findings can be attributed most parsimoniously to decision-stage factors governing both the speed and the precision of relative timing judgments.

We believe the task-specific RT ratios investigated here constitute a novel approach for exploring the factors that govern SJs and TOJs. Of course, there is a venerable line of reaction-time research on the so-called "redundant signals facilitation effect" [26–30]. In this effect, RTs can be hastened by presenting two stimuli within a temporal window of integration ("TWIN") [28, 29], an interval that resembles the SJ synchrony region (see caption for the present Fig 1). One recent study found this temporal window of integration to be wider for redundant-signals-facilitation than for SJs [29]. However, we have no knowledge of previous studies that described, predicted, and measured the RT ratios employed here to distinguish SJs from TOJs. These RT ratios controlled for consistent individual differences in overall reaction time, and for the reaction-time hastening that occurred across daily sessions (see Fig 8's right panels). Critically, the present RT ratios measure a construct–the hypothesized fluctuations in decisional uncertainty–distinct from the constructs indexed by PSS and temporal precision measures. Consequently, the fact that the present RT ratios distinguished SJs from TOJs constitutes a qualitatively new line of evidence that distinct factors govern these two relative-timing tasks. Notably, the present task-specific RT ratios and task-specific training effects on temporal precision occurred under visual stimulation identical to that in an earlier study that demonstrated distinct PSS values for SJs and TOJs [8]. Taken together, these three independent indices provide persuasive evidence that distinct factors govern SJs and TOJs.

Our results also extend previous reports that precision limitations can arise at physiological stages subsequent to stimulus-driven neural activity [31]. Recall that one draws this inference when training to improve the precision for a given stimulus feature fails to improve precision for a different feature of the trained stimulus. This task-specific learning–despite constant visual stimulation across tasks–has been demonstrated for a wide range of low-level feature discriminations. These include brightness versus orientation discrimination [31], local-element-orientation versus global-shape discrimination [32], speed versus direction discrimination [33], and simultaneity versus spatial frequency discrimination [34]. Our finding that training to improve SJ precision did not transfer to TOJ precision, and vice versa, suggests that the visual system may treat simultaneity and temporal order as distinct stimulus features. This seems remarkable given that SJ and TOJ precision could both depend, in principle, on a shared feature: the difference between target arrival times. Nature appears to have missed a “two-for-one sale”.

We note that the absence of training-transfer in the present measures of SJ and TOJ precision might be explained in at least two ways. One explanation comes from the task-specific decision boundaries, schematized in Fig 4. An alternative explanation would be captured by reweighting Fig 4‘s task-specific “connections” [20]. This possibility stems from the fact that SJs and TOJs have distinct informational requirements. SJs require *magnitude* information: the *absolute value* of the target-arrival-time difference. TOJs require *vector* information: the *sign* (positive versus negative) of the target-arrival-time difference. This suggests that distinct pooling strategies might optimize temporal precision for SJs and TOJs. For example, SJ precision might be optimized by pooling over (incorporating) neural activity that carries greater-than-chance information about target arrival time but questionable information about target-identity, i.e., "letter-target" versus "number-target" in the present study. By contrast, optimizing TOJ precision requires a strict correspondence between target arrival time *and* target identity. The loss of target identity impairs TOJs, but not SJs. In principle then, the observed lack of training-transfer in the present study may reflect these task-specific pooling strategies, rather than task-specific decision boundaries like those in Fig 4. Of course, Fig 4's task-specific decision boundaries correctly predicted the direction of the observed task-specific RT-ratios, and it remains unclear how these would arise from task-specific pooling strategies. Nevertheless, additional experiments will be needed to determine whether task-specific decision boundaries or task-specific pooling strategies better explain the limits on SJ and TOJ precision.

Further insights about temporal precision limits can arise from considering how the present SJ and TOJ perceptual learning findings in vision relate to those from the auditory modality. An earlier perceptual learning study measuring auditory evoked potentials revealed that practice-based improvements in auditory TOJs paralleled topographic changes in brain activity just 43–76 ms post stimulus onset [35]. The topography and brief latencies suggest that the initial stages of auditory processing had set the limit on auditory TOJ precision. This early-stage limit on auditory TOJs runs contrary to our findings on visual TOJs. Specifically, the lack of training-transfer observed in the present study–while stimulation remained identical across tasks–argues against an early-stage limit on visual TOJs. The discrepancy between the present (visual) and previous (auditory) perceptual learning studies demonstrates two points. First, the stimulus-driven stage sets limits on the precision of *some* temporal order judgments [35]. Consequently, as stimulation remained identical across the present TOJ and SJ tasks one might have plausibly expected training-transfer. The present lack of training-transfer was therefore not a foregone conclusion, and might even seem surprising. Conceivably, our training may have generated significant improvements in the stimulus-drive stage that were rendered undetectable (“masked”) by later visual stages containing prohibitively more imprecision. Stated differently, “connection” or “decision-related” stages (Fig 4) may have set the precision limit on our visual tasks, whereas the stimulus-driven stage may have set the precision limit in the previous auditory task [35]. This brings us to the second point; distinct rules may govern the temporal precision of auditory and visual judgments. Indeed, still other rules may govern the temporal precision of *audio-visual integration*, to which we now turn.

An apparent paradox arises when comparing the present findings to those obtained in a previous perceptual learning study on *audio-visual integration*. That study demonstrated that visual TOJ training significantly improved *audio-visual SJs* [36]. How can visual TOJ training fail to transfer to visual SJs (within a modality; present study), yet significantly *improve audio-visual SJs* (across modalities; [36])? This apparent paradox might be explained by considering, once more, what sets the limit on temporal precision. Notably, substantial evidence indicates coarser (worse) temporal precision in vision than in audition [37, 38]. As a salient example, human sensitivity to auditory interaural time differences (ITDs) occurs in the microseconds range [39] rather than the milliseconds range that typifies visual temporal precision. This orders-of-magnitude difference implies that, before training, audio-visual SJs in the previous perceptual learning study [36] more likely would have been limited by visual precision than by auditory precision. Accordingly, one possibility is that visual TOJ training improved audio-visual SJs “indirectly” by improving visual precision–the limiting factor in audio-visual precision. Stated differently, the previously reported training-transfer [36] might reflect a modality effect (vision versus audition) more so than task generalization (TOJs to SJs) *per se*. The same visual TOJ training would not necessarily be expected to improve the precision of visual SJs, particularly if distinct decision-related activity sets the temporal precision limit on visual SJs and visual TOJs. Overall, large baseline temporal-precision differences between vision and audition warrant caution when interpreting findings across these modalities.

We conclude with an admittedly speculative interpretation of the biological factors that may have mediated the present findings. One intriguing possibility comes from a visual attention study in which multiple surgically implanted electrodes simultaneously recorded parietal and prefrontal cortical activity [40]. The results revealed distinct neural activity patterns for top-down (task driven, “visual search”) and bottom-up (stimulus driven, “pop out”) attention. Specifically, the top-down attentional task generated comparatively lower-frequency (22–34 Hz) synchronous neural firing that began in the prefrontal cortex then later emerged in the parietal region. Contrariwise, the bottom-up attentional task generated comparatively higher-frequency (35–55 Hz) synchronous neural firing that began in the parietal region then later emerged in the prefrontal cortex. As a first approximation, these distinct activity patterns can be related to the framework schematized in Fig 4 of the present study. Specifically, one might envisage a parallel between prefrontal cortical activity [40] and the decision-related neural activity schematized in Fig 4‘s top boxes. Similarly, the stimulus-driven neural activity in Fig 4‘s bottom oval might parallel the previously measured parietal activity [40], and/or activity occurring still earlier in the visual pathway. Lastly, Fig 4‘s “Connection” arrows could reflect the previously observed [40] spread of synchronous neural firing between earlier (stimulus-driven, parietal) and later (task-driven, prefrontal) visual areas. To complete this analogy, the present task-specific RT ratios and absence of training-transfer would more strongly implicate prefrontal than parietal activity as the visual stage that sets the limit on SJ and TOJ precision. Likewise, the present findings would more strongly implicate the lower (22–34 Hz) than the higher (35–55 Hz) synchronous neural firing frequencies observed in the previous electrophysiological study [40]. Most importantly, we emphasize that these inferences are merely speculative. Further experiments are needed to determine the neural correlates of the novel task-specific RT ratios and training effects reported here.

## Supporting Information

S1 Data Raw Data for Reaction Time Analysis. (XLSX)

S2 Data Raw Data from SJ Training Group. (XLSX)

S3 Data Raw Data from TOJ Training Group. (XLSX)

S4 Data Raw Data from Control Groups. (XLSX)

S5 Data Raw Data From Participants Not Meeting Inclusion Criteria. (XLSX)

S1 List Participant List for Each Experiment. (XLSX)

S1 Movie Sample RSVP Movie. (MOV)
